# The Effects of Directional and Non-Directional Stimuli during a Visuomotor Task and Their Correlation with Reaction Time: An ERP Study

**DOI:** 10.3390/s23063143

**Published:** 2023-03-15

**Authors:** Francesca Miraglia, Chiara Pappalettera, Sara Di Ienno, Lorenzo Nucci, Alessia Cacciotti, Rosa Manenti, Elda Judica, Paolo Maria Rossini, Fabrizio Vecchio

**Affiliations:** 1 Brain Connectivity Laboratory, Department of Neuroscience and Neurorehabilitation, IRCCS San Raffaele Roma, 00166 Rome, Italy; 2 Department of Theoretical and Applied Sciences, eCampus University, 22060 Novedrate, Italy; 3 Neuropsychology Unit, IRCCS Istituto Centro San Giovanni di DioFatebenefratelli, 25125 Brescia, Italy; 4 Casa di Cura IGEA, Department of Neurorehabilitation Sciences, 20144 Milano, Italy

**Keywords:** EEG, ERP, reaction time, visuomotor task

## Abstract

Different visual stimuli can capture and shift attention into different directions. Few studies have explored differences in brain response due to directional (DS) and non-directional visual stimuli (nDS). To explore the latter, event-related potentials (ERP) and contingent negative variation (CNV) during a visuomotor task were evaluated in 19 adults. To examine the relation between task performance and ERPs, the participants were divided into faster (F) and slower (S) groups based on their reaction times (RTs). Moreover, to reveal ERP modulation within the same subject, each recording from the single participants was subdivided into F and S trials based on the specific RT. ERP latencies were analysed between conditions ((DS, nDS); (F, S subjects); (F, S trials)). Correlation was analysed between CNV and RTs. Our results reveal that the ERPs’ late components are modulated differently by DS and nDS conditions in terms of amplitude and location. Differences in ERP amplitude, location and latency, were also found according to subjects’ performance, i.e., between F and S subjects and trials. In addition, results show that the CNV slope is modulated by the directionality of the stimulus and contributes to motor performance. A better understanding of brain dynamics through ERPs could be useful to explain brain states in healthy subjects and to support diagnoses and personalized rehabilitation in patients with neurological diseases.

## 1. Introduction

During daily life, the visual attention is constantly captured by different stimuli indicating direction, such as road signs and symbolic arrow cues: the display of a pointing arrow should involuntarily induce—to some degree—a tendency in an observer to shift his attention to the indicated direction [1].

In the last two decades, previous studies have focused on comparing several types of “directional” stimuli based on gaze and arrow cues, by employing different methods of analysis, such as functional magnetic resonance imaging (fMRI) and electroencephalography (EEG) [2,3]. Hietanen and colleagues [4] measured blood oxygenation level-dependent (BOLD) signals using fMRI while participants performed visual tasks that included gaze and arrow cues, demonstrating that attention orienting by arrows depends on mechanisms connected to voluntary shifts of attention.

Although fMRI has been more widely used, EEG technology has an intrinsic higher temporal resolution, thus allowing researchers to study the timing of brain activations during a task [5]. Moreover, while fMRI signals arise from stimulus-related changes in energy consumption, one of the mechanisms for information flow via EEG modifications—namely transient, stimulus-related phase coherence and synchronization of the oscillating rhythms—does not require any energy consumption change and can therefore not be seen via fMRI [6]. In particular, one of the most popular techniques for investigating cognitive processes is to examine the EEG activity while time-locked to specific event of interest. In fact, a series of events generate the synchronization of a large group of characteristically firing neurons, which produce post synaptic activity at different cortical sites [7,8] revealed by EEG signal in event-related potentials (ERP) components [9,10,11].

ERPs are represented by a series of positive and negative polarity voltage deflections, related to a set of underlying components [12,13,14,15]. The latencies of ERP components track the time course of the processing activity; their amplitudes indicate the degree of distribution of neural sources necessary to support specific cognitive processes [16]. ERPs provide a method for studying attentional processes, language, and memory functions [11]; they are often used to study brain responses during visuomotor tasks [17,18].

Several studies have analysed ERP in response to cues that indicate a direction, observing that some typical components of attention orienting, voluntary control and maintenance of attention are involved firstly in the posterior parietal cortex, secondly in the lateral pre-frontal cortex and later on in the occipital–temporal region [19]. However, only a few studies explored differences in brain response due to directional and non-directional stimuli [20,21]. One of the first studies, published in 1995 by Wright and colleagues, has explored ERPs in response to both directional and non-directional cues during a spatial attention task, demonstrating that directional stimuli produce more focused attention and greater response preparation in terms of amplitude of P300 than non-directional cues. Hopf and collaborators (2000) revealed the presence of an initial component over the occipital-parietal scalp and an involvement of the posterior-parietal cortex in response to the directional cue, indicating the initial phases of attentional orienting. Hietanen and others (2008) have studied the networks of attention-orienting elicited by directional arrows cues using fMRI, revealing a large post-central and frontal eye-field activation, thus suggesting that attention orienting by arrow cues relies on mechanisms associated with voluntary shifts of attention. Brignani and collaborators [22] compared ERP components elicited by arrows, eye-gaze and simple neutral cues, demonstrating that the three types of stimuli induced a different strength of activation but within the same cortical networks, and suggesting different activation of the same neural networks. More specifically, the P1 showed the same posterior localization among the three conditions although arrows generated the smallest activation; the N1 also exhibited a posterior localization, but in this case arrows elicited a larger N1; finally, the longer-latency P3 (more similar to P3b) revealed a right centroparietal localization with a higher amplitude with textures. Moreover, rather than the neutral cue, the directional stimuli induced a faster attentional shift. Several studies demonstrated that the cover shift of attention that follows the cue presentation is associated with components in the ERP, which reflects the cortical processes involved in the control of visuospatial attention [19,23,24].

In this frame, our current research wanted to evaluate the ERP responses during a visuomotor task, in order to explore the differences in brain response to directional and non-directional cues immediately after the cue, as well as during the preparation of the motor response before a “go” stimulus.

In fact, the preparation for a “go” stimulus following the cue stimulus generates the contingent negative variation (CNV) [25,26,27]. The CNV is an event-related potential component which has been connected to different important cognitive processes as the preparation for the arrival of an upcoming event [28], the demanding of focused attention [29] and the timing of task execution [30,31]. In the current study, both early and late ERP components were investigated, aiming to describe the differences of focusing attention on the different types of cues (directional (DS) and non-directional (nDS) stimuli). We aimed to study the differences between the ERP components of participants’ performance as follows: firstly dividing them into two groups, the faster (F) and the slower (S) group, based on overall reaction times (RT); secondly dividing each participant’s recording into faster and slower trials based on the individual RT, in order to demonstrate, whether the amplitude of ERP components was influenced by the individual task performance.

Several previous ERP studies examined the relationship between ERP and performance, revealing some associations between RT and N200 and P300 latency, whereas fewer correlations were found with the earlier components (Coyle, Gordon, Howson & Meares, 1991; Iragui. Kutas. Mitchiner & Hillyard, 1993). Here, all the ERP components were evaluated in terms of amplitude and topographical distribution over the entire scalp, and in terms of latencies to reveal differences depending on the characteristics of the stimuli and on the performance of participants.

Accordingly, this study is presented as a deep analysis not only on the ERPs elicited by a visuomotor paradigm of attention orientation, but also on the preparation related to the directional or non-directional stimuli to the subsequent response to the “go” in terms of ERP components and reaction times (RT).

## 2. Materials and Methods

### 2.1. Participants

Experiments were performed on 19 healthy adult volunteers (9 females and 10 males, mean age = 26.05, standard error = 0.71). Based on the Handedness Questionnaire, all subjects were right-handed [32]. Their sight was normal or corrected-to-normal. Psychotropic or vasoactive drug therapy, and psychiatric or neurological conditions are listed in the exclusion criteria. All experiments were undertaken according to the ethical guidelines summarized in the 1964 Declaration of Helsinki and were approved by the local Ethical Committee. Informed consent was acquired from each subject before the beginning of the procedure.

### 2.2. Experimental Task

**Figure 1:**
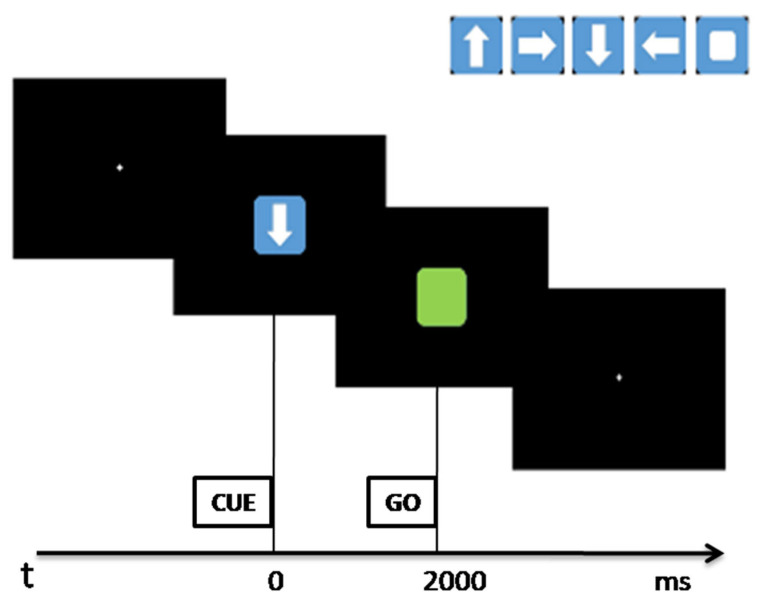
Typical trial of the experimental task: at T_0_ the cue stimulus appears; after 2000 ms, the go stimulus is presented. At the go stimulus, the subject has to respond as fast as possible, clicking the stimulus seen at the cue. In the top right of the figure the set of directional and non-directional kind of stimuli are reported.

The subjects were asked to sit in an armchair, in a dimly lit, sound-damped, and electrically shielded room. They were invited to keep their forearms on the armchairs and to position the right index finger on the numeric keypad on the “5” key. The monitor of the computer was located at a distance of around 60 cm. A typical trial was characterized by the sequence of visual stimuli shown in Figure 1 [33,34]: (1) a central white cross (diameter of 0.5°) in the background; (2) a cue stimulus as a square (size about 0.8°) with an “arrow” or a smaller “square” appearing inside, on the centre of the monitor for 2000 ms; (3) a go stimulus “green square” (size about 0.8°) lasting until subject response or until 2000 ms after its appearance; (4) the sequence restarting from point (1) for 2000 ms.

The cue stimuli at the centre of the monitor can be a white arrow on a blue square background, pointing to a direction (left, up, right, down), or a smaller white square on a blue square background meaning “no direction”. The cue was followed by a go stimulus that was a small green square in the centre of the monitor.

At the cue appearance, the participant was trained to only think about the direction presented by the stimulus, holding the index finger over the numeric keypad. Based on the direction of the cue stimulus, the subject had to press the numeric keypad at the corresponding key when the go stimulus appeared: “4” for left, “8” for up, “6” for right, “2” for down. In case of the white square appearing, the subject was free to choose a direction to think about, so to press the key indicating the chosen direction at go stimulus appearing. Each subject was instructed to maintain a fixed central finger position above the keys and to move exclusively to respond by pressing the correct key at the go stimulus appearance as soon as possible. The reaction times (RTs) to the go stimulus were recorded. In total, 400 pseudorandomised cues were presented (320 DS, 80 nDS) on a 24-inch monitor. The entire task was built using Presentation ^®^ software (Neurobehavioral Systems).

### 2.3. Data Recordings and Pre-processing Analysis

Electroencephalographic (EEG) signals were continuously recorded during the task from 64 electrodes (BrainAmp Brain Products) located in agreement with the International 10–20 scheme. Two distinct channels, horizontal and vertical EOGs, were used to check eye blinking. Impedance was maintained under the threshold of 5 kΩ and the sampling rate frequency was configured at 1000 Hz. The data were analysed in MATLAB, utilizing custom-made scripts built on the basis of EEGLAB functions (Swartz Center for Computational Neurosciences, La Jolla, CA, USA) [35,36]. In particular, the EEG data were resampled at 512 Hz and a band-pass finite impulse response (FIR) filter from 0.2 to 30 Hz was applied. EEG data were divided into single epochs for every single type of stimulus (S1, S2, S3, S4, S5, which refers to left, up, right, and down arrows and white square, respectively) of 5 s duration, from −1.5 s to +3.5 s around the onset of the cue stimulus. An EEG expert eliminated the main artifacts that appeared in the EEG recordings (such as eye movements, cardiac activity and scalp muscle contraction) initially by a visual inspection and then using Infomax procedure of Independent Component Analysis (ICA) as implemented in EEGLAB [37,38,39,40,41]. After the artifact’s removal process, no less than 60 epochs remained for each type of stimulus.

### 2.4. ERP Processing Analysis

Artifact-free EEG data were averaged separately for cue and go stimuli to compute ERPs. For both the cue and the go stimuli, EEG data were segmented offline into 1100 ms periods, starting 100 ms prior to specific stimulus onset and ending 1000 ms after the onset. Averages were computed relative to the 100 ms prior to the stimuli baseline and for each condition S1, S2, S3, S4 and S5 (i.e., left, up, right, and down arrows and white square, respectively). In general, the specific ERP’s components were selected as the largest peaks around the latency of interest and only time courses of specific channels were considered for the selection. In particular, the parietal and occipital electrodes P1/P2, P3/P4, P5/P6, P7/P8, PO3/PO4, PO7/PO8, O1/O2 and Pz/POz/Oz were selected for the early sensory components (P100, P150, N200) [20,42]; the central and parietal electrodes C1/C2, C3/C4, C5/C6, CP1/CP2, CP3/CP4, CP5/CP6, P1/P2, P3/P4, P5/P6, P7/P8 and Cz/CPz/Pz for the selection of the P300 component [43,44,45] and the central and parietal channels Cz/CPz were taken for the later components (P400) [46,47].

For the analysis of the contingent negative variation (CNV) to the go stimulus at 2000 ms, a baseline correction from 900 to 1000 ms was applied and the analysis was carried out on the vertex channel Cz [25,26]. For each subject, the ERPs on Cz in the range from 1000 ms to 2900 ms were extracted and the average between all the subjects was measured.

For each ERP component, the amplitude and latency values were assessed in the following statistical analysis.

### 2.5. Statistical Analysis

Statistical analyses were carried out in order to highlight different ERP responses which could be evoked by the vision of different stimuli: directional stimuli (DS) and non-directional stimuli (nDS), for both the cue and the go task interval. For the DS condition analysis, the four directional stimuli were considered all together (S1, S2, S3 and S4, which stand for left, up, right and down arrow, respectively), while for the nDS condition only the non-directional stimulus was studied (S5, which stands for the white square).

After checking the homogeneity of selected latencies between the conditions (DS, nDS) using Student’s *t*-test (*p* < 0.05), for both the cue and the go task intervals, two-tailed paired Student’s *t*-test analyses were conducted in order to evaluate the statistical differences between the P100, P150, N200, P300 and P400 components in the DS and nDS conditions.

Then, the subjects were divided into two groups, fast (F) and slow (S), in accordance with their reaction times (RTs) to the stimuli (for DS the mean time of the responses to S1, S2, S3 and S4, and for nDS to S5). Then, an unpaired *t*-test analysis was computed, for the go task interval, in order to assess the statistical differences between groups (F, S) and conditions (DS, nDS) in the P100, P150, N200, P300 and P400 components.

Specifically, after computing the mean RT for each subject, the median RT among all subjects and the mean RTs were evaluated. Then, the subjects with a lower mean RT than the median RT were included in the F group, while the subjects with a higher mean RT than the median one were included in the S group.

Moreover, in order to evaluate whether within each subject the faster and slower responses to the go stimulus could affect the ERP modulation, a further classification was performed. In particular, for each subject, the mean value and the standard deviation of the RT distribution were estimated in the DS (the number of epochs of nDS were not enough to compute ERP when they were divided into faster and slower groups for trials). Two groups of epochs were therefore obtained within each subject: the trials revealing a RT below the mean value (individually evaluated) minus half the standard deviation were classified as the group of fast (F) responses; the trials exhibiting an RT above the mean value plus half the standard deviation as the group of slow (S) responses. A paired *t*-test was computed to assess statistical differences between the groups of trials (F, S) for P100, P150, N200, P300 and P400 components.

We also analysed the collected components’ latencies in order to evaluate the differences in terms of ERP latency. In particular, an ANOVA was performed to investigate the difference between latencies for P100, P150, N200, P300 and P400 components between the conditions (DS, nDS). In addition, in order to test the differences between the groups (F, S), ANOVAs were computed for each latency of P100, P150, N200, P300 and P400 components and for each condition (DS, nDS). Finally, an ANOVA between trial groups (F, S) in the P100, P150, N200, P300 and P400 was performed.

Furthermore, Pearson’s linear correlation analysis was applied between the mean CNV values and the RTs, grouping all subjects together.

The statistical cut-off level of Student’s *t*-tests was fixed at *p* < 0.05. The significance levels of all Student’s *t*-tests were corrected using false discovery rate (FDR) correction to minimize the problem of multiple comparisons. In particular, FDR is the expected proportion of rejected hypotheses that are mistakenly rejected (i.e., the null hypothesis is actually true for those tests). Particularly, it returns a new threshold, instead of the starting threshold (alpha = 0.05), in order to minimize the Type I errors in multiple comparisons. After FDR, a new statistical cut-off level was obtained and reported.

## 3. Results

### 3.1. ERPs in Cue and Go Stimulus

An illustrative analysis of a representative subject was carried out to visualize the main event-related-potential components with the further aim of comparing the amplitudes of the ERPs between the cue stimulus (Figure 1A) and the go stimulus (Figure 1B). The key results highlighted an evident decrease in the amplitude of early components for the go stimulus rather than the cue target in every condition (DS, nDS), whereas an increase in later components was found. For both cue and go task intervals, each ERP component was carefully chosen after a visual inspection, selecting the specific channels mentioned above in which the component was supposed to appear, and the interval relative to the latency of interest.

In the first analysis, we explored the ERP responses using the view of the directional and non-directional stimuli (nDS), for both the cue and the go task interval.

**Figure 2:**
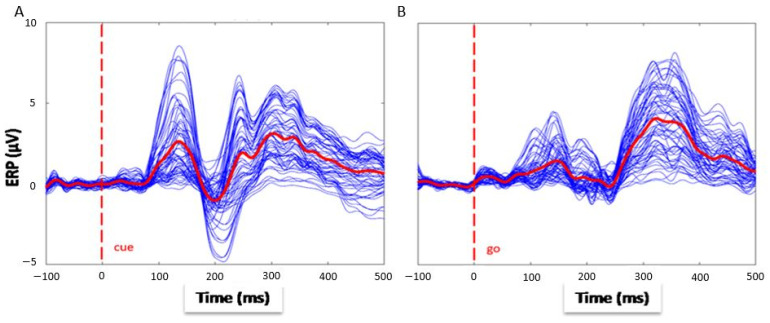
Butterfly plot of all channels and average trends (red lines) of ERP of a representative subject in the two conditions: the cue (**A**) and go (**B**) time intervals.

The key results highlighted an evident reduction/decrement in the amplitude of early components for the go stimulus rather than the cue target in every condition (DS, nDS), whereas an increase in later components was found. For both, cue and go task intervals, each ERP component was carefully chosen after a visual inspection, selecting the specific channels in which the component was supposed to appear, and the interval relative to the latency of interest. An illustrative analysis of a representative subject was carried out to visualize the main event-related-potential components to compare the amplitudes of the ERPs between the cue stimulus (Figure 2A) and the go stimulus (Figure 2B).

### 3.2. Directional Stimuli (DS) and Non-Directional Stimuli (nDS)

**Figure 3:**
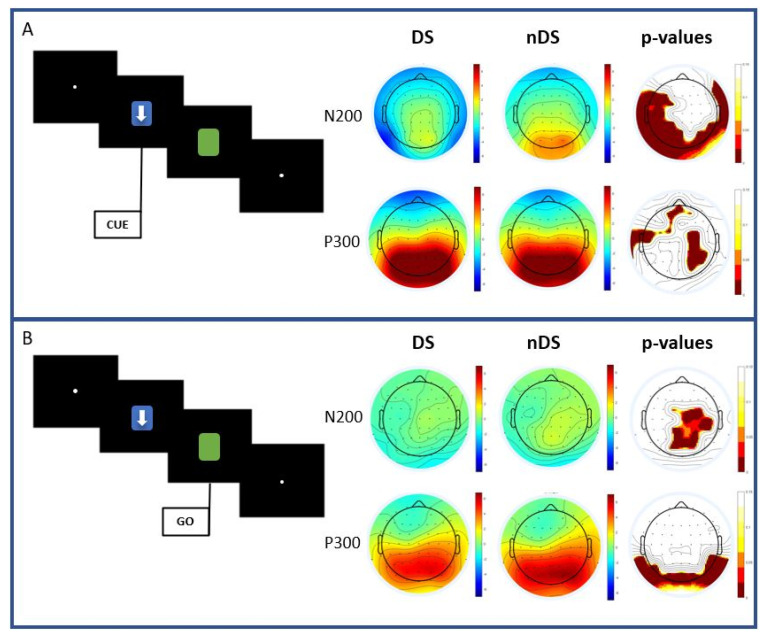
Mean scalp distribution of potentials in directional stimuli (DS) and non-directional stimuli conditions, and maps of statistical differences (*p*-value distribution) between DS and nDS in the cue (**A**) and go (**B**) stimulus for N200 and P300 latencies.

Based on the type of stimulus, the results showed no significant differences between DS and nDS conditions for the early components of P100 and P150. However, significant results were found in the N200 components; these were, in fact, more negative in amplitude in the DS compared to the nDS condition in the temporal, parietal and occipital areas, as shown in Figure 3A. Statistically significant differences were also obtained for the P300 component: here, smaller amplitudes in the central and temporal areas were found in the DS condition compared to the nDS one.

Regarding the go-stimulus analysis (Figure 3B), the current results showed no significant differences between DS and nDS conditions for the early components P100 and P150. Significant results were found in the N200, in which a more negative amplitude was found in the DS compared to the nDS condition in the central, parietal and occipital areas. Moreover, significant differences were found in the P300 component, where a smaller amplitude was found in the DS compared to the nDS condition in the occipital areas, as shown in the bottom part of Figure 3. The *p*-values, corrected using FDR, of statistically different results between DS and nDS are reported in Table 1 for each electrode. After the application of the FDR method, the new cut-off levels were *p* < 0.0445 for N200 and *p* < 0.0490 for P300 in the cue stimulus analyses, and *p* < 0.0455 for N200 and *p* < 0.0408 for P300 in the go stimulus.

**Figure 4:**
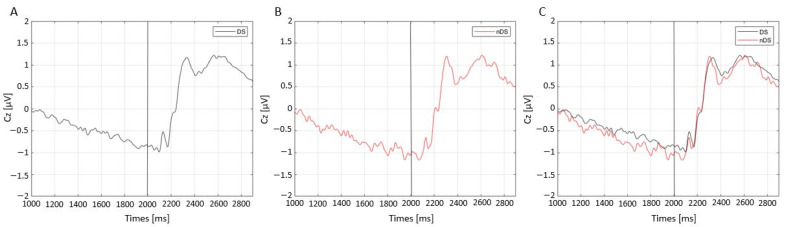
Contingent negative variation (CNV) trends of Cz electrodes after directional stimuli (DS) (**A**), non-directional stimuli (nDS) (**B**) and their overlap (**C**).

The CNV trends were plotted for both DS (Figure 4A) and nDS (Figure 4B) and the conditions (DS, nDS). In general, the results showed a smaller negative slope preceding the presentation of the imperative go stimulus (which occurs at 2000 ms) in the DS condition compared to the nDS one, as shown in Figure 4C.

### 3.3. Fast (F) and Slow (S) Groups in DS and nDS Conditions

In order to test the reaction time to the stimuli, the subjects were divided into two groups, fast (F) and slow (S), according to their reaction times (RTs) to the stimuli, for both the type of stimuli (cue and go) and conditions (DS, nDS).

**Figure 5:**
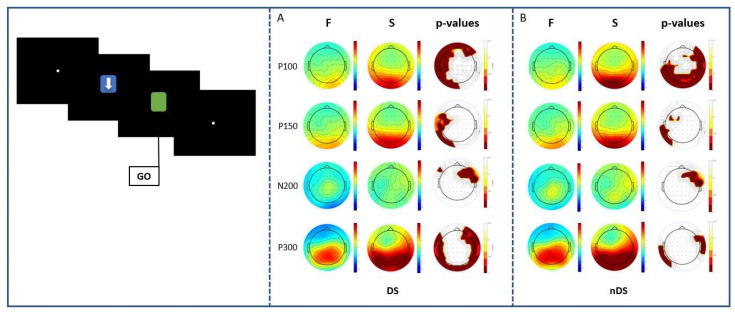
Mean scalp distribution of potentials in faster (F) and slower (S) groups and maps of statistical differences (*p*-value distribution) between F and S in the go stimulus of DS (**A**) and nDS (**B**) conditions for P100, P150, N200 and P300 latencies.

For the cue stimulus analysis, results showed no differences between fast (F) and slow (S) participants compared to RT in response to go, in both the DS and nDS conditions. However, for the go stimulus, significant differences were found in all the main component activations for both conditions (DS, nDS), as shown in Figure 5.

In particular, for the DS condition (Figure 5A), statistically significant differences were found in the P100 component: here, smaller amplitudes were reported in the F compared to the S group in the frontal and in the left parietal, temporal and occipital areas. Whereas in the P150 component, smaller amplitudes were reported in the left parietal and temporal areas in the F compared to the S group. Statistically significant differences were also found in the N200 component: results showed that the N200 is more negative in a limited part of the right frontocentral areas in F rather than in S subjects. Further significant differences were found in the P300 component on parietal, temporal and occipital areas; overall, a smaller amplitude of P300 was found in the F compared to the S group.

For the nDS condition (Figure 5B), significant differences were found in P100 and P150. In particular, a smaller amplitude of the P100 component was found in the F group compared to the S one in the parietal, temporal and occipital and areas. The same trend for the P150 component emerged in the left parietal areas. 

Furthermore, statistically significant differences were found in the N200 component mainly in the right frontocentral area, where a greater negativity was shown in the F group compared to the S one. For the P300 component, significances were found (shown as a smaller positivity) in the F compared to the S group in the temporal areas. In particular, the *p*-values of the electrodes, corrected using the FDR method, with mostly statistically significant differences are reported in the Table 2 for the DS condition and in Table 3 for the nDS condition. The new thresholds after the FDR correction are *p* < 0.0483 for P100, *p* < 0.0475 for P150, *p* < 0.0422 for N200 and *p* < 0.0441 for P300 in response to the DS, *p* < 0.0487 for P100, *p* < 0.0493 for P150, *p* < 0.0401 for N200 and *p* < 0.0494 for P300 in response to the nDS.

**Figure 6:**
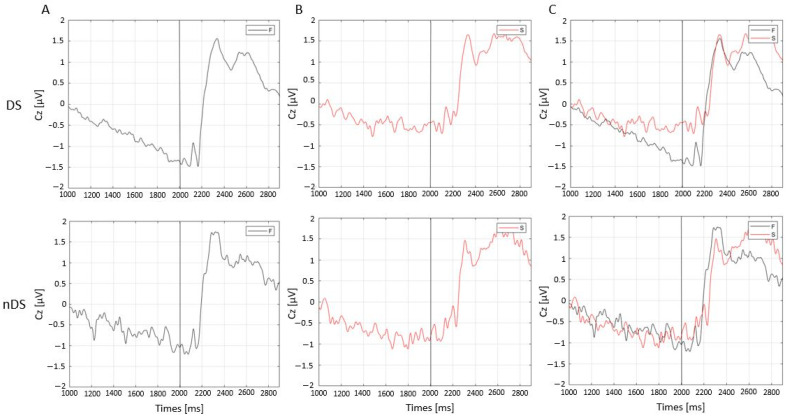
Contingent negative variation (CNV) trends of Cz electrodes in faster (F) (**A**) and slower (S) groups (**B**) and their overlaps (**C**) for directional (DS) (upper row) and non-directional stimuli (nDS) (lower row) conditions.

Regarding the CNV trends, the F group showed a clearly larger negative slope in preparation for the imperative go stimulus in the DS condition, whereas no differences were reported between the two groups in the nDS one (Figure 6).

### 3.4. Fast (F) and Slow (S) Trials

The same analysis was conducted between faster and slower trials extracted from all the subjects.

Moreover, in order to evaluate whether the faster and slower responses to the go stimulus could affect the modulation of ERPs, the analyses were conducted between faster and slower trials extracted from all the subjects.

**Figure 7:**
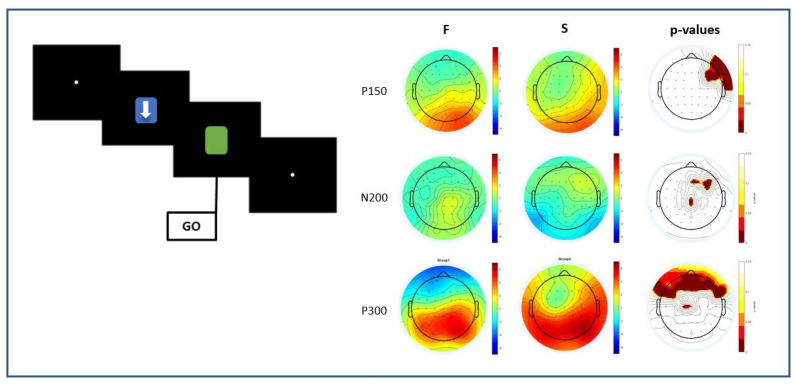
Mean scalp distribution of potentials in faster (F) and slower (S) groups of trials and maps of statistical differences (*p*-value distribution) between F and S in the go stimulus for P150, N200 and P300 latencies.

Significant differences were found at the latencies P150, N200 and P300. In detail, a strong statistical difference was found for the P150 component in the right frontotemporal area (*p* < 0.0483—corrected using FDR) where the component presented lower amplitudes in F compared to S trials. The N200 was less negative in the central area and more negative in the frontal area in F rather than in S trials (*p* < 0.0497—corrected using FDR). Further, a smaller amplitude of P300 was found in the F compared to the S groups of trials on the entire frontal areas (*p* < 0.0418—corrected using FDR) (Figure 7).

### 3.5. ERP Latencies

We also analysed the difference in latencies for P100, P150, N200, P300 and P400 components between the conditions (DS, nDS) and groups (F, S) with the aim of evaluating the differences in terms of ERPs’ latency.

The results did not show any differences between the DS and nDS conditions, while the ANOVAs applied to test the differences in latencies between the groups (F, S) separately for the two conditions (DS, nDS) showed that the S subjects had an earlier P300 latency compared to the S ones in the DS condition (*p* = 0.048).

Moreover, an ANOVA between trial groups (F, S) in the P100, P150, N200, P300 and P400 latencies was performed. Results between faster and slower trials within each subject showed that the faster trials presented an earlier modulation of the P300 (*p* = 0.005) compared to the slower ones.

### 3.6. Correlation Analysis

Pearson’s linear correlation analysis was performed between the mean CNV values and the RTs, grouping all subjects together.

**Figure 8:**
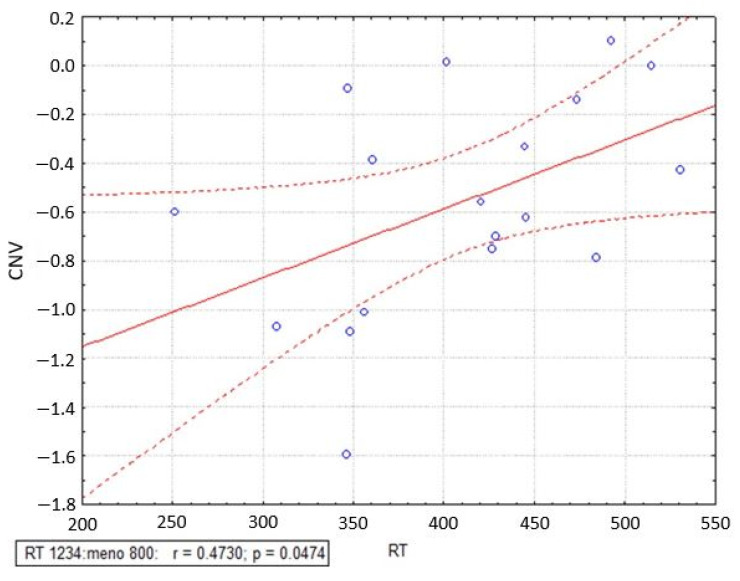
Correlation analysis between contingent negative variation (CNV) mean values and reaction times (RTs). The correlation line between the two types of data is represented as continuous while the correlation confidence intervals are shown as dashed lines.

Results showed a positive correlation (r = 0.0473, *p* = 0.0474) between RTs and mean CNV values (Figure 8), namely larger negative slope of the CNVs, the shorter the RTs and the faster the subjects.

## 4. Discussion

In healthy subjects, voluntary reorienting of attention guided by symbolic spatial cues is associated with different neural mechanisms [20,48]. The mechanism of visual attention is controlled by the dorsal posterior parietal cortex and the frontal cortex, which are implicated in the cognitive selection of sensory information and responses to that; and the temporoparietal and ventral frontal cortex employed during recognition of sensory experiences [49]. Our hypothesis is that directional stimuli might be processed differently compared to non-directional ones, showing differences in terms of amplitude and localization of event-related potential (ERP).

Therefore, the primary purpose of the current research was to analyse the differences between directional and non-directional stimuli in terms of ERPs during a visuomotor task, for both the cue and go presentations, which were followed by the immediate response of the subject.

The first findings revealed an evident modulation of ERP amplitudes from the cue to the go. Furthermore, for the cue condition in directional stimuli (DS) compared to non-DS (nDS) condition, a greater negativity of the N200 in the temporal, parietal and occipital areas was found, while the late components showed a lower amplitude of the P300 in the central and temporal areas. For the go condition, in DS compared to nDS stimulus presentation, significant results were found for the N200, in which a smaller amplitude was found in the DS compared to nDS condition in the central, parietal and occipital areas; and for the P300 component, where a lower positivity was notable in the occipital regions.

In line with our study, recent evidence suggested that in cue trials—where a response execution is not required—the early components show a greater amplitude (more negative for N200) due to a consequence of the inhibition of the response [50,51,52,53,54,55]. In the go trials, the later components show a greater amplitude of the ERP due to the overlapping of the motor response [56,57]. Notably, the two processes happen at the same time.

Moreover, in the analysis between the presentation of DS and nDS stimuli, the earlier components (P100 and P150) are purely visual components not linked to cognitive aspects: accordingly they did not show any differences because in the earlier milliseconds subjects could only see the cue stimulus without understanding what they were looking at [58,59,60]. The differences were evident in the later components when the subjects were able to detect and discriminate task-relevant targets. N200 latency is thought to reflect a process related to sensory discrimination of the stimuli [61]; the P300 reveals different cognitive processes of orienting, attention and working memory [62], and for attended stimuli as well [63]. The results suggested that the larger amplitude of N200 and the lower one for P300 derived from the greater mental load in the DS compared to the nDS that can involve cognition functions. Li and colleagues also suggested that the DS induces greater negativity in the N200 component than the nDS, because the stimuli containing directional cues provide more information, thus helping the brain understand the meaning more automatically and easily [64]. Regarding the go condition, the P300 showed the opposite trend, resulting in greater negativity in the nDS condition: the greater amplitude of P300 can be due to the greater overlapping of the motor response occurring in nDS trials [56,57].

In-depth study of the modulation of ERPs—especially late ERPs—can assist in the configuration of P300-based BCI systems for subjects to perform better when using them. Recent work [65,66] attempted to develop an optimal design of the P300-based BCI with visual stimulations, using photic [66] and vibrotactile [65] stimulations: they concluded that the investigation of the response to other types of stimuli (directional and non-directional) can introduce improvements in performance.

This result was confirmed by the CNV trend in the two conditions in the present study. In fact, for the preparation for the go stimulus, contingent negative variation (CNV) was computed, showing a lower slope for DS, indicating a faster preparation of participants to the go condition for the nDS.

The CNV slope, an indicator of motor preparation, is higher in nDS trials, suggesting a greater motor performance compared to DS trials. These results can be related to a lighter “free choice” mental load during the nDS tasks, whereas the DS trials require subjects to recall what the cues indicated.

The second part of the study explored a possible link between the preparation and the reaction to the go stimuli and the ERPs. The participants were firstly divided into two groups of “faster” (F) and “slower” (S) based on their mean reaction time to the go stimulus (RTs).

For the DS, in the F group lower values in the P100 and P150 in frontal and left central, parietal, temporal and occipital areas compared to the S group were found, while a greater negativity of the N200 in a limited part of right frontocentral area and a lower positivity of the P300 in parietal, temporal and occipital areas were found. For the nDS, the differences between F and S groups showed a great area of significance in temporal, parietal and occipital areas, revealing that the P100 of the F group has a lower amplitude compared to the S group. The same trend was obtained for the P150, although the area of difference was limited to the right parietal region. Additionally, the N200 was more negative in the F group in the frontocentral area and the P300 was less positive in the temporal regions.

The CNV trends between F and S groups in DS showed a greater slope for the F compared to the S group, indicating a faster preparation for the go stimulus of the F group only, whereas no differences were reported for the nDS.

As a further analysis, to depict whether the amplitude of ERP components was influenced by the individual task performance, each single participant’s recording was divided into F and S trials based on the individual RT. The maps showed the same topographical distribution of amplitudes compared to the previous analysis between F and S subjects. Concerning the analysis on ERP latencies, only the P300 was delayed both from F to S subjects, but also when F and S trials were analysed, demonstrating that a faster performance reflects an underlying process of a faster onset of the typical potentials occurring in response to a stimulus after 300 ms of latency. Finally, a positive correlation was observed among the CNV and the RTs, showing that the faster performers showed a greater slope of CNV, namely a better performance to the task.

Based on these findings, the P100 and the P150—previously described in adults in association with processing of visual stimuli—revealed a difference between the two groups: the current results confirm previous ones which demonstrated that greater P100 and P150 amplitudes are associated with longer reaction times, as in our case with “slower” subjects [67,68]. Concerning the N200, some studies demonstrated that more efficient responses to stimuli have been associated with increased negativity in the N200 amplitude [69], namely faster the subjects, the more negative the N200, as we have reported. Moreover, the modulation of the P300 could be explained as an elaboration of the stimuli at a different time: this might underlie the already present overlapping of motor response in the faster group, whereas the cognitive part is still present in the slower group [70]. Additionally, the attenuation of P300 can be due to more stressed performances [71]. Previous research has also demonstrated that when superior amounts of attentional reserves are required, P300 amplitudes appear to be reduced [72,73]. The lower amplitude of the P300 revealed a great difference in terms of latency; in particular, it was significantly delayed both in slower subjects and in slower trials, suggesting that a faster performance reflects an underlying process of a faster onset of the P300 in response to the stimulus. In fact, this component crucially reflects a processing speed of neurons [74] and an information cascade where attentional and memory processes are involved [45]. Moreover, P300 latency is supposed to determine the classification rate, which is proportional to the time necessary to identify and evaluate a target stimulus and is associated with mental functions. In a way shorter latencies are linked to enhanced cognitive performance [75,76]. Finally, comparing S and F groups, also in the present study, the slope of CNV reflects a different temporal integration [26], so the slower group reveals a slower drift of the CNV, while the faster one reveals a greater drift of the same components. In particular, this result is visible in the case of DS trials, while in nDS ones the CNV of the two groups overlapped. Most likely, the difference between the speed of the subject in the motor response is more evident when the task has a greater mental load, as in DS, while when the task requires a free-choice answer, as in the case of the nDS, the performances of the groups are essentially equivalent. The correlation analysis between the CNV slopes and the RTs confirmed these results, demonstrating that the greater CNV slope, the lower the RT, indicating a superior performance in the go stimulus response [25,77,78,79].

## 5. Conclusions

In conclusion, the current study highlighted activation of different ERPs after the presentation of directional and non-directional stimuli showing differences in term of amplitude and localization. The main findings are linked to the later components, when the subjects were able to detect and discriminate task-relevant targets, as the N200 reflects a process related to sensory discrimination of the stimuli and the P300 reveals different cognitive processes of orienting and attention.

Moreover, we observed that the ERPs were modulated by the performances, showing differences between fast and slow performers in both early and late components, and that the CNV slope is modulated by the directionality and contributes to the motor performance.

The novelty of the present study lies in the deep analysis of two aspects: first, the ERPs elicited by a visuomotor paradigm of attention orientation in terms of amplitude and topographical distribution over the entire scalp in terms of latencies; second, the preparation related to the directional or non-directional stimuli to the subsequent response to the “go” in terms of ERP components and reaction times (RT).

As the analysis of the ERPs in response to the visuomotor paradigm elicited some typical components of attention orienting, voluntary control and maintenance of attention, future studies could focus on the modulation of ERPs in an increased sample size, and on the motor performance such as in aging or in the rehabilitation of patients with motor deficits. This could allow finding biomarkers of functional recovery and plan personalized rehabilitation treatments. Moreover, in-depth study of the modulation of ERPs, especially late ERPs, can assist in developing an optimal design of the P300-based BCI systems [65,66].

## Figures and Tables

**Table 1:** Significant values (*p* < 0.05) related to all electrodes for N200 and P300 in the cue stimulus and the go stimulus between DS and nDS conditions. “-” indicates non-significant results. The results were corrected using the FDR method to resolve the issue of multiple comparisons.

	CUE		GO			CUE		GO	
	N200	P300	N200	P300		N200	P300	N200	P300
Fp1	-	-	-	-	C1	-	-	-	-
Fp2	-	-	-	-	C2	-	0.0034	0.0071	0.0034
F3	0.0217	-	-	-	P1	0.0183	-	0.0243	-
F4	-	-	-	-	P2	-	0.0124	0.0102	0.0124
C3	0.0038	-	-	-	AF3	-	0.0203	-	0.0203
C4	-	0.0014	0.0358	0.0014	AF4	-	0.0346	-	0.0346
P3	0.0045	-	-	-	FC3	0.0230	0.0429	-	-
P4	0.0154	0.0092	0.0389	0.0092	FC4	-	0.0019	0.0017	0.0019
O1	0.0002	-	-	-	CP3	0.0068	-	-	-
O2	0.0019	-	-	-	CP4	-	0.0018	0.0242	0.0018
F7	0.0043	-	-	-	PO3	0.0024	-	0.0406	-
F8	0.0327	-	-	-	PO4	0.0071	0.0487	0.0355	-
T7	0.0019	0.0475	-	-	F5	0.0191	-	-	-
T8	0.0109	-	-	-	F6	-	-	-	-
P7	0.0002	-	-	-	C5	0.0022	0.0346	-	0.0346
P8	0.0015	-	-	-	C6	-	-	0.0266	-
Fz	-	-	-	-	P5	0.0011	-	-	-
Cz	-	-	0.0396	-	P6	0.0049	0.0235		0.0235
Pz	-	-	0.0104	-	AF7	-	-	-	-
FC1	0.0401	0.0381	-	0.0381	AF8	-	-	-	-
FC2	-	0.0042	0.0016	0.0042	FT7	0.0056	0.0333	-	0.0333
CP1	0.0348	-	0.0396	-	FT8	0.0361	-	-	-
CP2	-	0.0098	0.0368	0.0098	TP7	0.0014	-	-	-
FC5	0.0056	0.0235	-	0.0235	TP8	0.0037	-	-	-
FC6	-	-	-	-	PO7	0.0002	-	-	-
CP5	0.0015	-	-	-	PO8	0.0016	-	-	-
CP6	0.0096	0.0385	0.0395	0.0385	Fpz	-	-	-	-
TP9	0.0001	0.0104	-	0.0104	CPz	-	-	0.0384	-
TP10	0.0028	-	-	-	POz	0.0070	-	0.0127	-
F1	-	0.0155	-	0.0155	Oz	0.0011	-	-	-
F2	-	-	-	-					

**Table 2:** Significant values (*p* < 0.05) related to all electrodes for P100, P150, N200 and P300 for the go stimulus in the directional (DS) condition between the fast (F) and slow (S) groups; “-” indicates non-significant results. The results were corrected using the FDR method to resolve the issue of multiple comparisons.

	DS					DS			
	P100	P150	N200	P300		P100	P150	N200	P300
Fp1	0.0231	-	-	-	C1	-	-	-	-
Fp2	0.0227	-	-	0.0382	C2	-	-	-	-
F3	0.0171	-	-	-	P1	-	-	-	-
F4	0.0437	-	0.0057	0.0274	P2	-	-	-	-
C3	0.0028	0.0229	-	-	AF3	0.0295	-	-	-
C4	-	-	-	-	AF4	0.0283	-	0.0274	0.0186
P3	-	-	-	-	FC3	0.0052	0.0037	-	-
P4	-	-	-	-	FC4	-	-	0.0075	0.0414
O1	0.0046	-	-	0.0178	CP3	0.0206	-	-	-
O2	-	-	-	0.0218	CP4	-	-	-	-
F7	0.0023	0.0290	0.0422	0.0289	PO3	-	-	-	-
F8	0.0281	-	0.0025	0.0043	PO4	-	-	-	-
T7	0.0001	0.0108	-	0.0048	F5	0.0033	0.0436	-	-
T8	-	-	0.0420	0.0016	F6	0.0398	-	0.0015	0.0092
P7	0.0009	0.0127	-	0.0112	C5	0.0007	0.0429	-	0.0441
P8	-	-	-	0.0078	C6	-	-	0.0253	0.0120
Fz	-	-	0.0348	-	P5	0.0176	-	-	0.0304
Cz	-	-	-	-	P6	-	-	-	-
Pz	-	-	-	-	AF7	0.0054	-	-	-
FC1	-	0.0474	-	-	AF8	0.0177	-	0.0080	0.0254
FC2	-	-	0.0400	-	FT7	0.0017	0.0340		0.0246
CP1	-	-	-	-	FT8	-	-	0.0038	0.0028
CP2	-	-	-	-	TP7	0.0001	0.0012	-	0.0023
FC5	0.0010	0.0088	-	0.0317	TP8	-	-	-	0.0035
FC6	-	-	0.0016	0.0035	PO7	0.0021	0.0258	-	0.0137
CP5	0.0020	0.0264	-	0.0158	PO8	-	-	-	0.0211
CP6	-	-	-	0.0346	Fpz	0.0225	-	-	-
TP9	0.0000	0.0017	-	0.0007	CPz	-	-	-	-
TP10	0.0147	0.0031	-	0.0019	POz	-	-	-	-
F1	0.0373	-	-	-	Oz	0.0235	-	-	0.0346
F2	-	-	0.0188	0.0385					

**Table 3:** Significant values (*p* < 0.05) related to all electrodes for P100, P150, N200 and P300 for the go stimulus in the non-directional (nDS) condition between the fast (F) and slow (S) group; “-” indicates non-significant results. The results were corrected using the FDR method to resolve the issue of multiple comparisons.

	nDS					nDS			
	P100	P150	N200	P300		P100	P150	N200	P300
Fp1	-	-	-	-	C1	0.0487	-	-	-
Fp2	-	-	-	-	C2	-	-	-	-
F3	-	-	-	-	P1	0.0134	-	-	-
F4	0.0350	-	0.0024	-	P2	0.0200	-	-	-
C3	0.0437	-	-	-	AF3	-	-	-	-
C4	-	-	-	-	AF4	-	-	-	-
P3	0.0156	-	-	-	FC3	-	0.0432	-	-
P4	0.0118	-	-	-	FC4	0.0149	-	0.0037	-
O1	0.0027	-	-	-	CP3	0.0139	-	-	-
O2	0.0182	-	-	-	CP4	0.0377	-	-	-
F7	-	-	-	-	PO3	0.0099	-	-	-
F8	0.0150	-	0.0219	0.0198	PO4	0.0216	-	-	-
T7	0.0069	0.0083	-	0.0331	F5	-	0.0488	-	-
T8	0.0032	-	0.0195	0.0289	F6	0.0193	-	0.0051	0.0337
P7	0.0011	0.0214	-	0.0471	C5	0.0343	-	-	-
P8	0.0052	-	-	-	C6	0.0107	-	0.0158	-
Fz	0.0350	-	0.0232	-	P5	0.0066	-	-	-
Cz	-	-	-	-	P6	0.0177	-	-	-
Pz	0.0212	-	-	-	AF7	-	-	-	-
FC1	-	0.0439	-	-	AF8	0.0342	-	0.0353	-
FC2	-	-	-	-	FT7	-	-	-	-
CP1	-	-	-	-	FT8	0.0042	-	0.0035	0.0235
CP2	-	-	-	-	TP7	0.0015	0.0039	-	0.0240
FC5	0.0347	0.0294	-	-	TP8	0.0052	-	-	0.0439
FC6	0.0063	-	0.0095	0.0494	PO7	0.0007	0.0232	-	0.0466
CP5	0.0025	0.0213	-	-	PO8	0.0119	-	-	-
CP6	0.0157	-	-	-	Fpz	-	-	-	-
TP9	0.0016	0.0019	-	0.0096	CPz	-	-	-	-
TP10	0.0044	0.0375	-	0.0185	POz	0.0191	-	-	-
F1	-	-	-	-	Oz	0.0072	-	-	-
F2	0.0095	-	0.0047	-					

## Data Availability

The datasets generated and/or analysed during the current study are available from the corresponding author on reasonable request.
